# The making of eusociality: insights from two bumblebee genomes

**DOI:** 10.1186/s13059-015-0635-z

**Published:** 2015-04-24

**Authors:** Romain Libbrecht, Laurent Keller

**Affiliations:** Department of Ecology and Evolution, University of Lausanne, CH-1015 Lausanne, Switzerland; Rockefeller University, 1230 York Avenue, New York, NY 10065 USA

## Abstract

The genomes of two bumblebee species characterized by a lower level of sociality than ants and honeybees provide new insights into the origin and evolution of insect societies.

## Bumblebees enter the genomic era

In 1995, John Maynard Smith and Eörs Szathmáry classified the transition from solitary to social life as one of the major transitions in evolution, along with the transitions from prokaryotes to eukaryotes, and from asexual to sexual reproduction [1]. The highest level of social organization is found in eusocial insects (for example, ants, termites and some bees and wasps) that are characterized by societies with overlapping generations, cooperative brood care and the coexistence of fertile queens and functionally sterile workers [2]. It is common to differentiate two categories of eusociality: primitive eusociality, where queens are morphologically similar to workers, and advanced eusociality, where queens and workers follow a different developmental pathway, resulting in marked differences between adults.

Since the publication of the genome of the honeybee *Apis mellifera* in 2006, the genomes of two other honeybee and nine ant species have been sequenced. These valuable genomic resources provided insights into several key features of eusocial Hymenoptera, including the developmental processes leading to queen and worker caste differentiation, division of labor, chemical communication and immunity [3]. However, because honeybees and ants show advanced eusociality, it has so far not been possible to investigate the molecular changes associated with the early stages of the evolution of social life.

In this issue of Genome Biology, Sadd and colleagues [4] and Barribeau and colleagues [5] present and analyze the genomes of two bumblebee species - *Bombus terrestris* and *Bombus impatiens*. These two primitively eusocial species are particularly interesting because they share a primitively eusocial ancestor with the honeybees.

## The comparison of the honeybee and bumblebee genomes

The honeybees and bumblebees belong to the Apidae, a group of insects that are important pollinators and possess a similar diet. However, these taxa exhibit marked differences in their life history and social organization. Honeybee colonies are perennial, typically containing tens of thousands of workers that cannot mate and are morphologically different from the queen heading the colonies. By contrast, bumblebees have a yearly cycle, with an overwintering queen starting a colony in spring. By the end of the season, when colonies can contain up to a few hundreds of workers at most, new queens and males are produced. Bumblebee workers are usually smaller than the queen, but they are not morphologically different, and they can, in principle, mate.

The comparison of the genomes of *B. terrestris* and *B. impatiens* revealed strong similarity. In particular, the genetic architecture is very conserved, with high synteny and less than 10 chromosomal rearrangements. This is striking because these two species diverged 18 million years ago, and bumblebees are known for their high rate of recombination. The genomes of the honeybee and bumblebees were also found to share many similarities. They are all characterized by a low number and low diversity of repetitive elements, similar developmental gene repertoire, biogenic amines and neuropeptide suite, a reduced set of detoxification enzymes, a similar number of odorant and ionotropic receptor genes, and a similar DNA methylation machinery.

Despite these similarities, fine differences exist between the honeybee and bumblebees. Bumblebees show a recent expansion of gustatory receptor genes and a change in domain repeat numbers for proteins that have functions related to muscles, which could be associated with differences between honeybees and bumblebees in taste detection and muscle use during flight, respectively. The bumblebee genome contains two genes coding for proteins that bind to juvenile hormone (JH) that do not have orthologs in the honeybee. This finding is interesting because the JH signaling pathway plays a key role in regulating division of labor in eusocial Hymenoptera. Known to affect reproduction in solitary insects, JH might have been co-opted to regulate the behavior of sterile workers in advanced eusocial species such as the honeybee. Finally, there were many more microRNAs in the honeybee than in the bumblebees. This is exciting because microRNAs are involved in the regulation of many aspects of the honeybee social life. Whether those differences are actually associated with advanced eusociality remains to be investigated.

## Social and individual immunity in bees

Insect societies are usually characterized by high population density, frequent social contacts and high relatedness, which should make them particularly good targets for the spread of pathogens. However, eusocial insects have evolved a suite of collective defenses, known as social immunity, that comprise both prophylactic and activated measures to protect themselves against pathogens. Such measures include mechanical removal of parasites by grooming, avoidance of infected individuals, waste management and any behavior enhancing nest hygiene in general [6]. A famous example is found in wood ants that incorporate conifer resin into their nest, thus using the antimicrobial properties of the resin as a means of defense against pathogenic microorganisms [7].

The sequencing of the honeybee genome revealed a low number of immunity genes compared with the number present in the fly and mosquito genomes. This led earlier authors to suggest that social immunity might have decreased the selective pressure on individual immunity, resulting in less immunity genes either by gene loss or limited gene duplication. While this hypothesis has been supported by the finding of a similarly small immune gene repertoire in ant genomes, it has later been challenged by further genomic studies. First, a recent study showed no evidence for relaxed selection (as expected if social immunity had reduced the selection pressure) on the immune gene repertoire in honeybees and ants [8]. Second, the genomes of the solitary wasp *Nasonia vitripennis* and the solitary pea aphid *Acyrtosiphon pisum* also have a low number of immune genes, suggesting an expansion of the immune gene repertoire in flies and mosquitoes rather than a reduction in the eusocial honeybees and ants [9]. According to this hypothesis, a small repertoire of immune genes would be ancestral to eusociality.

The genomes of the bumblebees provided Barribeau and colleagues [5] with the opportunity to test this prediction in the bee lineage. They compared the sets of immunity genes across the advanced eusocial honeybee, the intermediate eusocial bumblebee and a solitary bee that diverged from the ancestor of honeybees and bumblebees before it evolved eusociality. As expected, if the low number of immune genes was the ancestral state, Barribeau *et al.* found comparable low numbers of immunity genes, irrespective of the complexity of social organization. Thus, this result shows that, in the bee lineage, the limited number of immunity genes is not a consequence of, but rather predates, the evolution of eusociality.

## Concluding remarks

Exciting times lie ahead for biologists interested in the origin of eusociality, which evolved at least 10 times in insects. There are a minimum of nine independent origins of eusociality in Hymenoptera (five times in different bee lineages, three times in different wasp lineages and once in ants) and one in Isoptera (which gave rise to termites). The recent sequencing of a termite genome [10], as well as the imminent publication of a wasp genome, give the opportunity to study two additional origins of eusociality in insects. It has been almost a decade since the publication of the first eusocial insect genome, and each additional sequenced genome has been another step towards unveiling the making of eusociality.

## Abbreviation

JH: Juvenile hormone
